# Aurora Medical Society

**Published:** 1880-12

**Authors:** 


### ARTICLE VIII.

AT a meeting of the Aurora Medical Society, held at Aurora, Ill., November 8th, a specimen was presented and the following history given by Dr. M. M. Robbins.

Was called about four o'clock p. m., October 22, 1880, to see J. B. W., a machinist, aged 57, who had been wounded by a pistol ball. Found him laboring under severe shock. Under the use of stimulants, he rallied, reaction on the following day being excessive.

On examination, assisted by Dr. E. B. Howell, the ball was found to have entered the left arm at a point about five inches below the shoulder joint, emerging about two inches below the axillary fold, passing anterior to the bone and not injuring it. It then struck the chest over the fifth rib, passed inwards and slightly downwards to the fourth costal interspace, and a little inside a line drawn perpendicularly from the left nipple. Beyond this point the probe would not pass. He improved quite rapidly. He had no pain, except from a bronchitis, which he had contracted previous to the injury. There were no symptoms indicating that the ball had injured any vital organ.

On the eighth day after injury, he was dressed. On the ninth day he was out of doors. On the tenth he walked about still more. On the eleventh he walked about home a short time in the morning. About ten o'clock he took a short ride in an easy carriage; returned home; said that he felt better than at any time since the injury. During the afternoon he walked about or rested upon the sofa, as he felt inclined; ate a light supper about 6:30 p. m.; chatted for awhile after eating. Said frequently that except being a little weak, he felt better than at any time since his injury. He walked from the table to one corner of the room, sat down, and immediately complained of a severe pain and pressure in his chest, in the cardiac region. He walked to another part of the room, and in a few minutes died. It was estimated by different members of his family that he lived from three to eight minutes after the serious symptoms began—probably not more than the shorter time. He had lived eleven days, three and one-half hours after the injury.

Post mortem fourteen hours after death. Present Drs. Higgins and E. B. Howell.

On opening the chest, a considerable quantity of blood was found in the left pleural cavity, and the pericardium was filled with blood. About one-half of that in the pericardium was in the form of a recent clot. No old clot could be found. The ball had struck the upper border of the fifth rib, at the point to which we had traced it at the time of injury, described nearly a right angle with its previous course, perforated the pericardium, struck the left ventricle upon its anterior surface about two-fifths of the distance from the base to the apex, passed into and through that cavity nearly horizontally, and lodged in its posterior wall, through which it nearly passed at the time of injury. At this point there was a stellate rupture. This was at the apex of a slight elevation upon the surface, the walls of which were very thin. It is possible that these walls had been thinned by being worn by the roughened surface of the ball after its lodgment there.

There were old adhesions of the pleural surfaces at the left of the heart, which accounted for the pain in that region which attended his cough. One of the aortic valves was ossified. This was the only lesion referable to the heart discovered before death, although careful examination was made for any symptom which would indicate that the heart had been injured. The ball was of 32 caliber. I am told that he ran about fifty feet after receiving the wound before he became unconscious.

The wounds in the arm, chest and anterior wall of the ventricle were entirely healed. One of the pillars of the columnæ carneæ was located immediately over the point where the ball entered the ventricular cavity, and may possibly have prevented or helped to prevent the flow of blood through the wound.

